# The Role of Genetic Testing in Pediatric Renal Diseases: Diagnostic, Prognostic, and Social Implications

**DOI:** 10.7759/cureus.44490

**Published:** 2023-08-31

**Authors:** Sultan A Alharbi, Abduljabbar M Alshenqiti, Ali H Asiri, Musaed A Alqarni, Saad A Alqahtani

**Affiliations:** 1 Department of Pediatrics, Prince Mohammed Bin Abdulaziz Hospital, Madinah, SAU; 2 Department of Pediatrics, Khamis Mushait Maternity and Children Hospital, Khamis Mushait, SAU; 3 Department of Pediatrics, King Salman Hospital, Riyadh, SAU

**Keywords:** test, genetic, disease, renal, pediatric

## Abstract

Pediatric renal diseases vary widely and are linked to high morbidity and mortality; hence, early diagnosis is vital. Presently, genetic testing is being incorporated into the standard of care for children and their families with kidney disease, primarily as a diagnostic tool. In the present review, we aim to collect all potential evidence from relevant studies that reported the role of genetic testing in pediatric renal disease diagnostic, prognostic, and social implications. We have conducted both electronic and manual searches within PubMed, the Cochrane Library, Web of Science, and Scopus to find relevant studies. Studies from the years 2013-2023 were included. Case reports with limited sample sizes and no descriptive statistics, along with review papers and meta-analyses, were excluded from this review. Quality assessment for all included studies was performed. The pooled diagnostic yields were calculated using the common effect and random effect models utilizing the R program (R Foundation for Statistical Computing, Vienna, Austria). The pooled result for the diagnostic yield as per the common effect model is a pooled proportion of 0.42 (42%) 95% confidence interval (CI): [0.39,0.44], while with the random effects model the pooled proportion is 0.43 (43%) 95% CI: [0.31,0.57]. The diagnostic yield for the included studies ranged from 78.10% to 16.8%. The spectrum of kidney diseases included nephrolithiasis/nephrocalcinosis, glomerular diseases, cystic kidney disease, ciliopathies, tubulopathies, chronic kidney disease, and congenital anomalies of the kidneys and urinary tracts (CAKUT), while hematuria and proteinuria were reported by two studies and autosomal recessive and autosomal dominant idiopathic kidney disease was reported by only one study. Genetic testing validates clinical diagnosis and aids in tailoring management strategies; hence, a more precise treatment plan is developed and unnecessary investigations are avoided, which is crucial in the case of children during routine nephrology clinic visits. Genetic counselling is of the utmost importance, so all ethical and social concerns related to genetic testing are addressed in addition to patient satisfaction.

## Introduction and background

Pediatric kidney diseases include a wide range of disease entities with varying clinical manifestations, courses of development, and treatment choices. Children with end-stage renal disease constitute a greater portion of the monogenic condition population, accounting for almost 30% of children with chronic kidney disease (CKD) [1]. Globally, pediatric CKD has a severe impact and is linked to high cardiovascular and all-cause death rates, as well as significant morbidity. Pediatric CKD can be asymptomatic until it reaches an advanced stage. A delay in CKD diagnosis can result in morbidity that is preventable and increased healthcare expenses [2]. The global prevalence of CKD is reported to be 15-74.7 cases per million children [3]. CKD exhibits distinct characteristics in children and can, at least in part, be thought of as a separate nosologic entity. Additionally, some common characteristics of pediatric CKD, such as the etiology of the condition or cardiovascular issues, may not only affect the child's health but also have a long-term effect on their adult life [4].

Kidney disease often goes undiagnosed in the general population, but due to the nature of the causes of the disease and the ambiguity of the symptoms, children and adolescents are at an even higher risk. Younger pediatric patients with renal diseases may have nonspecific clinical manifestations unrelated to the urinary system [5]. Children with urological anomalies that may be curable illnesses are frequently referred to after the disease has progressed. It can be challenging to make an early diagnosis of renal diseases in hospitalized children and young adults since they often exhibit few symptoms at first, progress differently from adults, and have a variety of therapeutic responses [5]. The spectrum of renal diseases varies significantly, and even within the same nation, there are regional variations in the pattern of childhood renal disease. Genetic predisposition, environmental background, and, to a significant extent, level of consciousness all have an impact on this diversity. In contrast to industrialized nations, the causes are different in developing nations. In general, 4.5%-8.7% of all pediatric admissions are due to pediatric renal disease. Kidney disease in children admitted to hospitals is frequently misdiagnosed. Undiagnosed fever or failure to thrive may be the only symptoms during infancy and early childhood [6].

In addition to approximately 50% of children and 10% of adults who enroll in end-stage renal failure programs, it is predicted that 20% of children with renal disease may have an underlying genetic malformation. Gene identification for a range of renal disorders is made possible by massively parallel sequencing, which has become an essential part of renal research. More recently, the creation of approved diagnostic testing platforms has made it possible to integrate sequencing technology into routine clinical practice. The families of children with renal disease can benefit from information provided by genetic testing, such as the opportunity for an earlier diagnosis, the identification of relatives who are at risk, and possibly a decrease in morbidity and mortality. However, the difficulty of variant interpretation is increased by the high genetic variability of these disorders and the ongoing validation of new genes and/or novel variations. Rapidly, genomics is being incorporated into the standard of care for children and their families with kidney disease, primarily as a diagnostic tool but also to guide therapy [7]. The use of next-generation sequencing techniques has greatly increased the diagnostic yield in patients with inherited kidney disorders, according to findings of numerous groundbreaking studies [1,3,4]. In the present review, we aim to collect all potential evidence from relevant studies that reported the role of genetic testing in pediatric renal disease diagnostic, prognostic, and social implications.

## Review

Methods

Definition of Outcomes and Inclusion Criteria

Our objective was to assess and evaluate the role of genetic testing in the diagnosis and prognosis of renal diseases among children and discuss the social implications associated with it. Consequently, we included original research studies that discussed the spectrum of kidney diseases diagnosed through genetic testing in children. All studies with participants aged less than 18 years were included, while review articles and meta-analyses were excluded. Moreover, case reports with limited sample sizes and no descriptive statistics were also excluded from this review. Other exclusion criteria were nonhuman or laboratory studies, nonoriginal investigations or incomplete studies, abstract-only articles, protocols, theses, and articles that were not published in English or with no available English information. In addition, seven articles were pooled for meta-analysis to determine the diagnostic yield.

Search Strategy

After obtaining our desired outcomes, we performed a brief manual screening of potentially included studies to identify relevant keywords for the most appropriate search term. Our search term (pediatrics OR infants OR children) AND (renal disease OR kidney disease OR nephropathy) AND (genetic testing OR DNA testing OR genetic screening OR genetic predisposition testing OR genetic predictive testing) AND (diagnosis OR examination OR assessment) AND (prognostic factors OR prognosis AND social implications OR social impact OR social consequences OR social effect). The databases for the search included PubMed, the Cochrane Library, Web of Science, and Scopus. Our search was restricted to the title and abstract of the search results to ensure we included all relevant studies. All results were then saved to an Endnote library, where we identified and removed duplicates across the different databases. Additionally, we manually searched the reference lists of the included studies and relevant reviews, as well as similar article sections in PubMed, to identify any missed studies by our electronic search strategy. We followed the Preferred Reporting Items for Systematic Reviews and Meta-Analyses (PRISMA) guidelines throughout all stages of this systematic review.

Screening and Extraction

To ensure the accuracy and quality of our review process, we implemented a double screening strategy, which involved screening both titles/abstracts and full texts. Two reviewers conducted the screening process in a blinded manner, and a senior member evaluated the entire process and facilitated discussions among the reviewers in case of discrepancies. We constructed an extraction sheet that was organized in a manner relevant to our research objectives, which included baseline characteristics, publication details, abstracts, decisions to include or exclude articles, and the reasons for exclusion. We also identified whether each study was a clinical trial or not. We made sure to include all relevant articles that met our inclusion criteria.

Quality Assessment

We extracted information from the included studies regarding the potential risk of bias in these studies. To assess the quality of observational studies, we used the QUADS-2 scale, which comprises primarily two domains: risk of bias and applicability concerns, which further consist of sub-domains including patient selection, index testing, follow-up, timing, and reference standards. Studies were graded from high to low based on the degree of bias.

Statistical Analysis

The statistical analysis was performed using the R software (R Foundation for Statistical Computing, Vienna, Austria), employing the meta package. The pooled diagnostic yield was estimated using both the common effect model and the random effect model.

Results

Search Results

**Figure 1:**
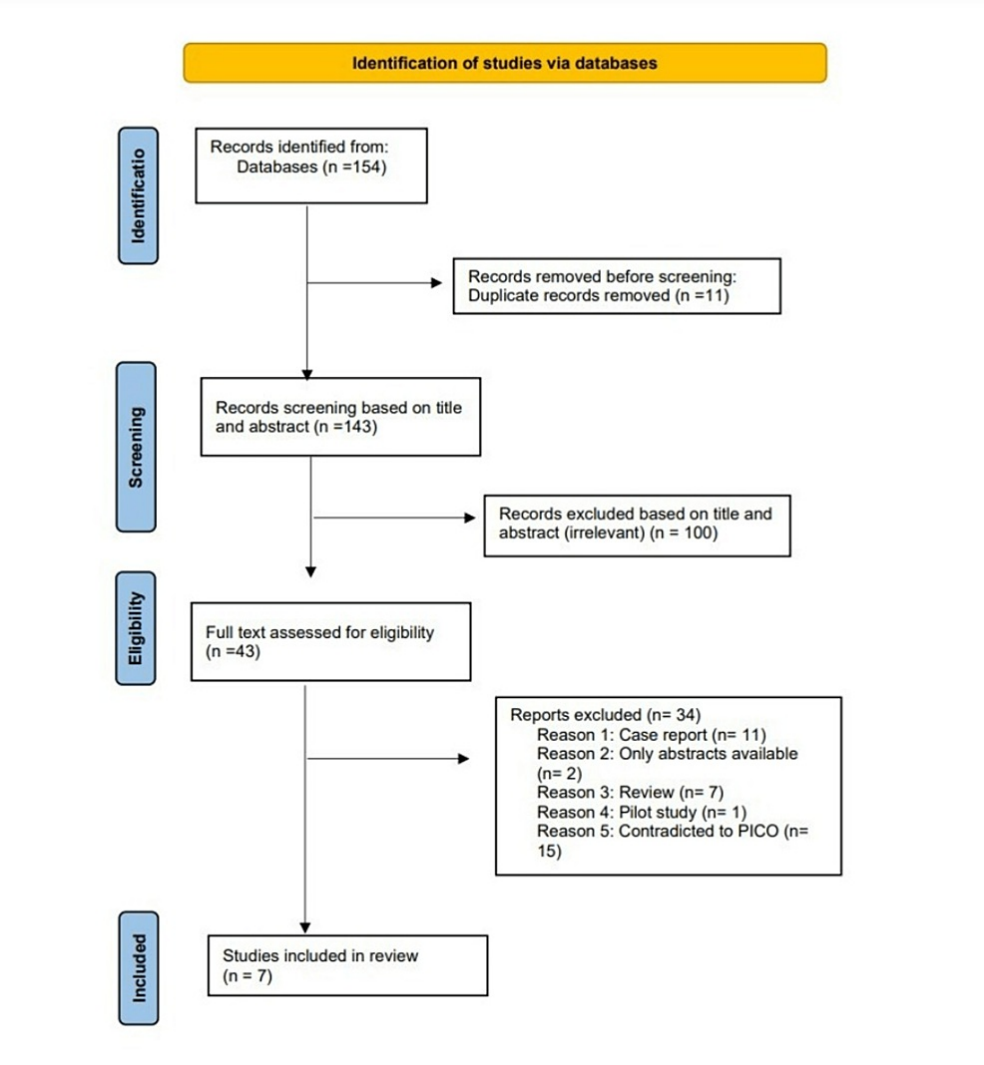
Preferred Reporting Items for Systematic Reviews and Meta-Analyses (PRISMA) Flow chart

We conducted the search strategies as described above and identified a total of 154 citations, which were then reduced to 143 after removing duplicates. After screening titles and abstracts, only 43 citations were considered eligible for the next steps. Full-text screening narrowed down the number of articles to seven that matched our inclusion and exclusion criteria. Figure 1 shows the detailed search strategy and screening process.

Results of Quality Assessment

The quality assessment of the included studies revealed that the majority of studies had good quality and satisfactory results with a low risk of bias. The reference standard was Low for all the included studies. Detailed results of the quality assessment according to the QUADS-2 scale are illustrated in Table 1.

**Table 1:** Quality Assessment – QUADS-2

Study	Risk of Bias				Applicability Concerns		
	Patient Selection	Index Test	Reference Standard	Follow and Timing	Patient Selection	Index Test	Reference Standard
Huang et al., 2022 [8]	Unclear + yes + yes = unclear	No + no = High	Yes + yes+ yes = Low	Yes + yes + yes + yes = Low	Low	Low	Low
Chen et al., 2023 [9]	Unclear + yes + unclear = unclear	No + yes = unclear	Unclear + unclear + yes = unclear	Yes + yes + yes + yes = Low	Low	Low	Low
Bekheirnia et al., 2021 [10]	Unclear +yes + yes = unclear	No + no = High	Yes + yes + yes = Low	Unclear + yes + yes + no= unclear	Low	unclear	Low
Rao et al., 2019 [11]	Yes + yes + yes = Low	No + yes = unclear	Yes + yes + yes = Low	no + yes + yes + yes = unclear	Low	Low	Low
Gao et al., 2023 [12]	Unclear + yes + yes+ = unclear	No + yes = unclear	No + no + yes = unclear	No + yes + yes + yes = unclear	Low	Low	Low
Vaisitti et al., 2023 [1]	Yes + yes + yes = Low	No + no = High	Unclear + yes + yes = unclear	No + yes + yes + yes = unclear	Low	Low	Low
Braun et al., 2016 [13]	No + yes + no = unclear	No + yes = unclear	yes + yes + yes = Low	No + yes + yes + yes = unclear	Low	Low	Low

Characteristics of the Included Studies

We included seven studies that recruited 1841 patients and were published between 2013 and 2023. The total population consisted of 59.3% males and 40.6% females. All the included studies were observational studies, with the majority being cohort studies and two retrospective studies. Regarding the geographical distribution of the included studies, the majority were from China, followed by two studies from the United States of America, among which one also included a European cohort, and one study from Italy. All the baseline characteristics of these studies are shown in Table 2. There are variations in the sample size of included papers, which are likely due to the objective of the specific study, the design, and the inclusion criteria.

**Table 2:** Baseline characteristics of included studies

Author	Country	Year	Study design	Total participants	Mean age	Gender Male/Female%
Rao et al., 2019 [11]	China	2019	cohort	1001	Median age 5 years	59.74%/40.26%
Bekheirnia et al., 2021 [10]	USA	2021	Retrospective cross-sectional study	192	Mean 8.7 ± 6.0	53%/47%
Chen et al., 2023 [9]	China	2023	Retrospective cohort	133	Median (range): 5 years (4 months -14 years)	66.17%/33.83%
Gao et al., 2023 [12]	China	2023	cohort	149	Median (range): 3 years (4 days- 13 years)	63.8%/36.2%
Vaisitti et al., 2023 [1]	Italy	2023	cohort	191	<18 years	57%/43%
Huang et al., 2022 [8]	China	2022	Retrospective study	32	ranging in age from 3 months to 14 years	65.6%/34.4%
Braun et al., 2016 [13]	United States and Europe	2016	cohort	143	<18 years	50.3%/49.7%

Study Outcome Measures

**Figure 2:**
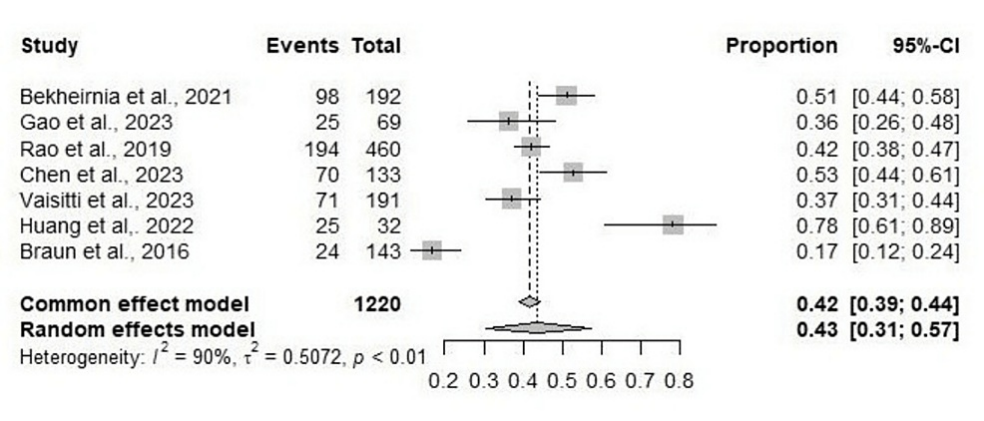
Meta-Analysis: Pooled Diagnostic Yield and Confidence Intervals

The pooled result for the diagnostic yield as per the common effect model is a pooled proportion of 0.42 (42%), 95% confidence interval (CI): [0.39,0.44], while with the random effect model, the pooled proportion is 0.43 (43%), 95% CI: [0.31,0.57] (Figure 2).

Huang et al. reported 40.6% cases of nephrolithiasis, 40.6% of nephrocalcinosis, and 18.8% cases of nephrolithiasis+nephrocalcinosis with the highest total diagnostic yield of 78.1% among the included studies [8], followed by Chen et al., who reported a total diagnostic yield of 52%, while the spectrum of kidney diseases showed 28.87% cases of isolated hematuria, 39.1% of proteinuria and hematuria, 10.53% of simple proteinuria, 13.53% of renal insufficiency, and 8.27% of others [9]. Bekheirnia et al. stated quite a closer total diagnostic yield of 51% with 26% cases of cystic kidney disease, 21% of congenital anomalies of the kidneys and urinary tracts (CAKUT), 20% of hematuria, and 11% of proteinuria [10].

Rao et al. reported 55.3% of cases of glomerular disease; CAKUT accounted for 15.9% of cases; 8.3% of cystic renal disease; 15.9% of renal tubular disease and renal calcinosis or nephrolithiasis; and 4.6% of CKD 3-5 stages, while the total diagnostic yield was 42.10% [11]. Gao et al. described that 41.8% of cases accounted for X-linked inherited kidney disease, while for both autosomal dominant and autosomal recessive inherited kidney disease, there were 29.1% of cases each, and the total diagnostic yield was 36.3% [12]. Vaisitti et al. reported that glomerular diseases accounted for 32.5%, ciliopathies for 20.4%, CAKUT for 17.8%, nephrolithiasis for 11.5%, tubular disease for 10.5%, and 7.3% for other diseases, and the total diagnostic yield was 37.1% [1]. Braun et al. demonstrated 86.01% cases of nephrolithiasis and 13.99% cases of nephrocalcinosis, while the total diagnostic yield was 16.8% [13].

The diagnostic yields within the subgroups among the included studies are as follows: Huang et al. revealed 69.2% for nephrolithiasis, 84.6% for nephrocalcinosis, and 83.3% for nephrolithiasis+nephrocalcinosis, while the mutated genes were CLCN5 and OCRL [8]. Chen et al. reported 44.74% for isolated hematuria, 59.62% for proteinuria and hematuria, 50% for simple proteinuria, 50% for renal insufficiency, and 54.55% for others. The mutated genes observed were ACE, ADCK4, COL4A4, COL4A5, CLCN5, PKD1, and SMARCAL1 [9]. The diagnostic yield for the Bekheirnia et al. study showed 42% for CAKUT, 79% for cystic kidney disease, 70% for proteinuria, and 67% for hematuria, and the observed mutated genes were COL4A4, HNF1B, PKD1, PKHD1, and WT1 [10]. In Rao et al. the diagnostic yield was 29.1% for glomelular diseases, 61.4% for cystic renal disease 17% for CAKUT, 62.3% for renal tubular disease/renal calcinosis and 23.9% for CKD [11]. The mutated genes in Gao et al. study were COL4A3, COL4A4, COL4A5, CLCN5, NPHS1, PKD1, PKHD1, SMARCAL1, OCRL and others [12]. In a study by Vaisitti et al., the yield was 20.6% for CAKUT, 74.4% for ciliopathies, 24.2% for glomerulopathies, 45.5% for nephrolithiasis, 45% for tubulopathies, and 7.1% for others [1]. Braun et al. reported a diagnostic yield of 13% for nephrolithiasis and 40% for nephrocalcinosis with OCRL as a mutated gene [13]. Results in detail are exhibited in Tables 3, 4, 5.

**Table 3:** Outcome measures of included studies CAKUT: congenital anomalies of the kidneys and urinary tracts; CKD: chronic kidney disease; IKD: inherited kidney diseases; SRNS: steroid-resistant nephrotic syndrome

Study	Total diagnostic yield	Subgroups	Diagnostic yield of subgroups
Bekheirnia et al., 2021 [10]	51%	Cystic kidney disease 26%, CAKUT 21%, Other 23%, Hematuria 20%, Proteinuria 11%	CAKUT 42%, Cystic kidney disease 79%, Proteinuria 70%, Hematuria 67%
Gao et al., 2023 [12]	36.30%	Autosomal dominant IKDs 29.1%, Autosomal recessive IKD 29.1%, X-linked IKDs 41.8%	
Rao et al., 2019 [11]	42.10%	Glomerular disease (SRNS, nephritis, aHUS) 55.3%, CAKUT 15.9%, Cystic renal disease (Nephrolithiasis, PKD, multicystic renal dysplasia) 8.3%, Renal tubular disease and renal calcinosis or nephrolithiasis/stone 15.9%, CKD 3 - 5 stage with unknown origin 4.6%	SRNS 29.1%, Cystic renal disease 61.4%, CAKUT 17.0%, Renal tubular disease/renal calcinosis 62.3%, CKD 3 to 5 stage with unknown origin 23.9%.
Chen et al., 2023 [9]	52.63%	Isolated hematuria 28.57%, Proteinuria and hematuria 39.1%, Simple proteinuria 10.53%, Renal insufficiency 13.53%, Other 8.27%	Isolated hematuria 44.74%, Proteinuria and hematuria 59.62%, Simple proteinuria 50%, Renal insufficiency 50%, Other 54.55%
Vaisitti et al., 2023 [1]	37.10%	Glomerular diseases 32.5%, Ciliopathies 20.4%, CAKUT 17.8%, Nephrolithiasis 11.5%, Tubular disease 10.5%, Other 7.3%	CAKUT 20.6%, Ciliopathies 74.4%, Glomerulopathies 24.2%, Nephrolithiasis 45.5%, Tubulopathies 45%, Others 7.1%
Huang et al., 2022 [8]	78.10%	Nephrolithiasis 40.6%, Nephrocalcinosis 40.6%, Nephrolithiasis+Nephrocalcinosis 18.8%	Nephrolithiasis 69.2%, Nephrocalcinosis 84.6%, Nephrolithiasis+Nephrocalcinosis 83.3%
Braun et al., 2016 [13]	16.80%	Nephrolithiasis 86.01%, Nephrocalcinosis 13.99%	Nephrolithiasis 13%, Nephrocalcinosis 40%

**Table 4:** List of commonly mutated genes NA: not available

Study	Commonly mutated genes										
	ACE	ADCK4	COL4A3	COL4A4	COL4A5	CLCN5	HNF1B	NPHS1	PKD1	PKD2	PKHD1
Bekheirnia et al., 2021 [10]	NA	NA	NA	4.90%	17.30%	NA	4.90%	NA	18.50%	NA	4.90%
Gao et al., 2023 [12]	NA	NA	14%	9%	35%	4%	NA	7%	5%	NA	7%
Rao et al., 2019 [11]	NA	NA	NA	NA	NA	NA	NA	NA	NA	NA	NA
Chen et al., 2023 (9)	3.45%	8.05%	NA	14.94%	26.44%	3.45%	NA	NA	3.45%	NA	NA
Vaisitti et al., 2023 [1]	NA	NA	NA	NA	NA	NA	NA	NA	NA	NA	NA
Huang et al,. 2022 [8]	NA	NA	NA	NA	NA	6.30%	NA	NA	NA	NA	NA
Braun et al., 2016 [13]	NA	NA	NA	NA	NA	NA	NA	NA	NA	NA	NA

**Table 5:** List of commonly mutated genes NA: not available

Study	Commonly mutated genes												
	QCRL	SMARCAL1	WT1	Other	OCRL	SLC4A1	KMT2D	HOGA1	SLC3A1	CYP24A1	ADCY10	SLC34A1	VDR
Bekheirnia et al., 2021 [10]	NA	NA	4.90%	NA	NA	NA	NA	NA	NA	NA	NA	NA	NA
Gao et al., 2023 [12]	NA	4%	NA	11%	4%	NA	NA	NA	NA	NA	NA	NA	NA
Rao et al., 2019 [11]	NA	NA	NA	NA	NA	NA	NA	NA	NA	NA	NA	NA	NA
Chen et al., 2023 [9]	NA	3.45%	NA	NA	NA	NA	NA	NA	NA	NA	NA	NA	NA
Vaisitti et al., 2023 [1]	NA	NA	NA	NA	NA	NA	NA	NA	NA	NA	NA	NA	NA
Huang et al,. 2022 [8]	NA	NA	NA	NA	6.30%	9.40%	9.40%	6.30%	NA	NA	NA	NA	NA
Braun et al., 2016 [13]	NA	NA	NA	NA	8.33%	NA	NA	NA	12.50%	8.33%	8.33%	29.20%	8.33%

Discussion

Over the past two decades, revolutionary advancements in genetic knowledge and technology have radically changed the understanding of pediatric renal disorders. Such advancement is starting to have a significant effect on normal clinical management as well as diagnostic and prognostic evaluation of juvenile renal disorders. The main causes of CKD in children are CAKUT and genetic conditions that affect specific nephron components. More than 160 genes, including those involved in nephrogenesis, primary cilia development and function, podocyte and tubular cell activities, complement regulation, and more, have been linked to hereditary nephropathies to date. A genetic diagnosis can be made in 15%-20% of cases of severe kidney malformations, up to 30% of cases of steroid-resistant nephrotic syndrome, 60%-70% of cases of complement-mediated atypical hemolytic uraemic syndrome, and 50-80% of cases of hereditary tubulopathy following a systematic screening of all known genes within each disease group by next-generation sequencing [14].

**Figure 3:**
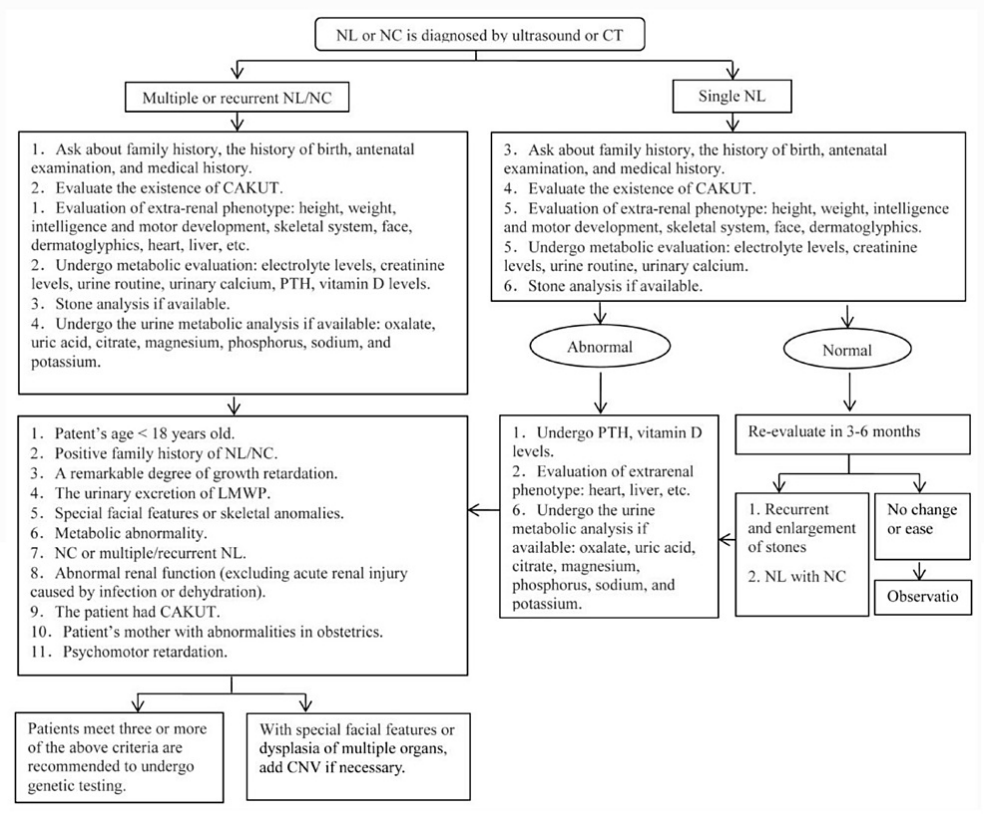
Strategies for performing genetic testing in nephrolithiasis and nephrocalcinosis [8]. NL: nephrolithiasis, NC: nephrocalcinosis, PTH: parathyroid hormone, CAKUT: congenital anomalies of kidney and urinary tract, LMWP: low molecular weight protein

Our results demonstrated that cases of nephrolithiasis and nephrocalcinosis were reported by four of the included studies, with the total diagnostic yield ranging from 42.10% to 16.8%. Nephrolithiasis and nephrocalcinosis have a complex etiology that includes heredity, systemic illnesses such as inflammatory bowel disease, parathyroid dysfunction, metabolic variables, anatomical abnormalities of the kidneys, and recurrent urinary tract infections [15], although the primary causes of nephrolithiasis and nephrocalcinosis are hereditary factors [8]. There have been reports of more than 30 genes being connected to the monogenic types of nephrolithiasis and nephrocalcinosis. As there is an almost deterministic cause-effect relationship in monogenic disease, the ability to identify the underlying mutations in a monogenic disease gene is of significant diagnostic and maybe therapeutic relevance [16,17]. The criteria and strategies of gene testing for nephrolithiasis and nephrocalcinosis by Huang et al. are illustrated in Figure 3.

Groopman et al. described that when no pathogenic variant is discovered in the molecular diagnostic tests for individuals who meet three or more of the aforementioned inclusion criteria, it is imperative to give attention to the possibility of genetic disorders. Therefore, it is advised to give careful consideration to the clinical phenotype, follow up on the family history, and employ more modern molecular diagnostic procedures, such as whole genome sequencing, next-generation sequencing, and chromosome copy number variation, if necessary [18]. Moreover, Johnson et al. narrated that all children with nephrolithiasis and nephrocalcinosis require a thorough evaluation with clinical phenotype due to the high risk of recurrence and potential unfavorable prognosis, and genetic testing must be done in patients with a high risk of hereditary diseases in order to provide genetic counselling and appropriate management. As a result, reliable and thorough information about phenotype and family history is required to interpret the results of molecular diagnostic tests [19].

Furthermore, the advantages of genetic testing in case of nephrolithiasis and nephrocalcinosis include the utilization of the results of genetic testing for developing a molecular diagnosis, direct treatment to halt the development of stones and calcification, and offering genetic counselling. Genetic testing may be useful when the clinical phenotype is insufficient for diagnosis but inherited renal tubular disease is strongly suspected [8]. Additionally, the relevance of genetic diagnosis in children cannot be overstated for various reasons. The first is that it might be significant in terms of how the disease is treated clinically; a typical example of this is with nephrotic syndromes, where the identification of structural variants in genes related to podocytes provides evidence against immunosuppressive treatments that would otherwise be regularly administered over a period of several months [20].

Secondly, it might be very important for the family of the child and for identifying other family members who have the mutation that might be passed on to future generations. Cascade testing of family members and genetic counselling for variant carriers are routine procedures in clinical genetics if a harmful variant is discovered in a proband [21]. Thirdly, in the scenario of transplantation, where the donor may be a relative, understanding the harmful variation is crucial. It is crucial to identify all family members who might require a transplant as well as rule out the possibility that the organ donor carries the same variants. Furthermore, some disorders, such as focal segmental glomerulosclerosis, have a significant chance of relapsing after organ transplantation, or a more specialized selection of the transplant to be carried out may enhance their prognosis, like in the case of primary hyperoxaluria, where a combined kidney-liver transplant may produce a better prognosis [22,23]. For the patient to participate in clinical trials and have access to cutting-edge treatment alternatives, a precise disease diagnosis may be helpful [24,25].

The pooled proportion of diagnostic yield for this study was 50.55% (common effects model) and 42.03% (random effects model). The diagnostic yield for the included studies ranged from 78.10% to 16.8%. Domingo-Gallego et al. reported a global diagnostic yield of 65% [26], which is higher than our results, but this difference may be due to the inclusion of the adult population in their study. Gefen et al. concluded that with definite, probable, or potential explanatory variations discovered in up to one-third of children with nephrolithiasis/nephrocalcinosis, genetic testing had a high yield and has the potential to advance therapeutic practice. Rapid, inexpensive genetic testing has made it easier to diagnose genetic renal disease [27]. De Haan et al. described that genetic testing may improve the diagnostic precision in CKD patients, particularly in those whose etiology is uncertain. The diagnostic value of next-generation sequencing has primarily been demonstrated in cohorts of children with CKD. Next-generation sequencing can aid in the diagnosis of kidney disorders with unusual presentations, where it may result in reclassification of the primary renal disease diagnosis, in addition to its implications for unexplained CKD [28].

Arora et al. reported that more than 450 monogenic diseases have been identified as the cause of chronic kidney disease to date; these disorders account for 5-30% of cases in adult cohorts and over 30% of cases in pediatric cohorts. The previous Sanger sequencing technique involved serially studying individual genes but was costly and time-consuming. It was replaced by the next-generation sequencing technique, which made it possible to examine several genes simultaneously and swiftly produce results. Next-generation sequencing was initially employed to investigate a group of genes that caused a disorder with a particular phenotype, such as Alport syndrome and others. Whole-exome sequencing was utilized to find novel genes. About 33,000 exons from all 22,000 genes, or about 1-2% of the protein-coding regions, were able to be studied with the assistance of whole exome sequencing. This technique of analysis continues to be the basis for the identification of novel genes involved in a variety of renal illnesses. To accurately study these molecular alterations, which frequently occur deep within introns or as a result of copy number variations, whole genome sequencing, which examines both introns and exons, is required [29]. Various kidney diseases among children are demonstrated in our findings which were diagnosed by genetic testing. Additionally, the predominant mutations were in the genes COL4A4, CLCN5, PKD1, and PKHD1.

Other predominant renal diseases in addition to nephrolithiasis and nephrocalcinosis observed in our findings include glomerular diseases, cystic kidney disease, ciliopathies, tubulopathies, CKD, and CAKUT, while heaturia and proteinuria were reported by two studies and autosomal recessive and autosomal dominant idiopathic kidney disease was reported by only one study. Ahn et al. narrated that since 1995, when a mutation in PAX2 was originally identified as the cause of optic nerve coloboma, renal hypoplasia, and vesicoureteral reflux, genetic causes of CAKUT have been identified. More than 40 genetic abnormalities have been found in earlier research, and more than 50 genes have been linked to CAKUT. Currently, monogenic causes, the majority of which have a dominant pattern of inheritance, can account for up to 18% of CAKUT patients. Further research utilising chromosomal microarrays revealed that 4.5-16.6% of CAKUT patients have genetic abnormalities, particularly those with renal hypodysplasia. Most copy number variants that cause CAKUT have previously been linked to various developmental abnormalities, including neurocognitive problems, heart anomalies, and developmental delays. A genetic diagnosis of CAKUT enables family assessment, genetic counselling, accurate diagnosis of the severity of the condition, and evaluation of patients for extrarenal symptoms [30].

Furthermore, understanding the genetic, epigenetic, and environmental roots of kidney and urinary tract abnormalities is crucial from a therapeutic standpoint because CAKUT is the leading cause of kidney failure in children. This area of the genomic landscape of CAKUT has been extensively delineated by the advent of next-generation sequencing. Currently, more than 50 genes linked to the aetiology of CAKUT have been discovered. Although the development of next-generation sequencing and bioinformatic methods has enhanced knowledge of the molecular landscape of CAKUT, the majority of the patients' aetiologies are still unknown. To better describe the molecular architecture of CAKUT, the integration of extensive human genetic, epigenetic, and environmental investigations is therefore vital. Clinical decisions will include a more accurate assessment of CAKUT risk factors and complications, as well as realistic projections of kidney function survival and overall prognosis, when the results of such studies are combined with diagnostic imaging studies and biochemical parameters of disease progression [31]. Additionally, Sweeney and Avener described that it is possible to have genetic testing for both autosomal dominant and autosomal recessive polycystic kidney diseases. In vitro fertilization and preimplantation genetic testing are now being provided by specialized clinics to parents who have already had an affected child [32].

Social implications

The fields of ethics, public health, and genetics have come together as a result of the rise in genetic testing and concerns of discrimination by insurance companies, employers, and society as a result of genetic testing. It is critically necessary to consider whether the family of someone who has a positive predictive genetic test should be made aware of the findings and hazards. The moral and ethical responsibilities of the patient and the diagnosing clinician must be discussed. Depending on the moral theory being applied, deciding whether or not to disclose will change. The notions of ethics and beneficence will also be significant factors in the choice [33].

The etiology and mechanics of kidney disorders have become much clearer owing to growing genetic understanding, but ethical concerns threaten to thwart the practical application of genomic medicine. The expansion of genetic research holds enormous promise for enhancing the treatment of people with heritable kidney disorders and their families. However, families, physicians, and researchers face significant ethical challenges as a result of this kind of research and associated practices [34]. In the past, genetic kidney disease was frequently identified when family members had similar clinical characteristics. Currently, a pathogenic mutation of a gene linked to the disease is used to diagnose many hereditary renal diseases. When a genetic mutation is identified, the method of inheritance and potential family members are also revealed. In general, genetic testing requires informed consent because the outcome offers certainty with ramifications for the patient, their family, and possibly their work, as well as having social, ethical, and economic impacts. Genetic testing should also be made available to any of their at-risk relatives who are accessible. Patients who consent to the sharing of their anonymized registry results speed up the diagnosis process for other families and contribute to a better understanding of these diseases for everybody [35].

It is normal practice to test and screen children's genetics. The best interests of the children should be taken into consideration while deciding whether to give genetic testing and screening. Best practices can be influenced by the rising body of research on the clinical and psychosocial impacts of such testing and screening. Parents or guardians should be advised to disclose genetic test findings to their children at an appropriate age. However, they should also be aware that mature adolescents usually have the right to request their own test results. It's crucial to note that these results may affect not only the child but also the family, leading to possible family stress, delays in receiving results, implications for further family planning, and the cost of testing. Timely and appropriate counseling is essential, and adequate human resources for such counseling may be a significant barrier. When appropriate, healthcare professionals have a duty to discuss these potential implications with parents and the child [36]. The systematic search methodology and the analysis of all keywords in this field are this study's main advantages and strength. However, the publication period and exclusion of review papers, in addition to considering only English papers, are limitations of this study. Furthermore, the results of our study could not be compared more elaborately with the findings of other studies in the literature, which is due to the dearth of studies in the literature and the inclusion of only the pediatric population, which necessitates the need for further research in this aspect.

## Conclusions

Genetic testing validates clinical diagnosis and aids in tailoring management strategies; hence, a more precise treatment plan is developed and unnecessary investigations are avoided, which is crucial in the case of children. The findings of our study have further highlighted the role of genetic testing for the diagnosis of kidney diseases among children, especially during routine nephrology clinic visits. However, during routine nephrology clinic visits, genetic counselling is of utmost importance, so all ethical and social concerns are catered for in addition to patient satisfaction.
